# VUV to IR Emission Spectroscopy and Interferometry Diagnostics for the European Shock Tube for High-Enthalpy Research

**DOI:** 10.3390/s23136027

**Published:** 2023-06-29

**Authors:** Ricardo Grosso Ferreira, Bernardo Brotas Carvalho, Luís Lemos Alves, Bruno Gonçalves, Victor Fernandez Villace, Lionel Marraffa, Mário Lino da Silva

**Affiliations:** 1 Instituto de Plasmas e Fusão Nuclear, Instituto Superior Técnico, Universidade de Lisboa, 1049-001 Lisbon, Portugal; ricardojoaogmferreira@tecnico.ulisboa.pt (R.G.F.); bernardo.carvalho@tecnico.ulisboa.pt (B.B.C.); llalves@tecnico.ulisboa.pt (L.L.A.); bruno@ipfn.tecnico.ulisboa.pt (B.G.); lionel.marraffa@tecnico.ulisboa.pt (L.M.); 2 European Space Agency—European Space Research and Technology Centre, 2201 AZ Noordwijk, The Netherlands; victor.fernandez.villace@esa.int

**Keywords:** atmospheric entry, shock tube, streak camera, vacuum ultraviolet, visible, infrared, microwave interferometry

## Abstract

The European Shock Tube for High-Enthalpy Research is a new state-of-the-art facility, tailored for the reproduction of spacecraft planetary entries in support of future European exploration missions, developed by an international consortium led by Instituto de Plasmas e Fusão Nuclear and funded by the European Space Agency. Deployed state-of-the-art diagnostics include vacuum-ultraviolet to ultraviolet, visible, and mid-infrared optical spectroscopy setups, and a microwave interferometry setup. This work examines the specifications and requirements for high-speed flow measurements, and discusses the design choices for the main diagnostics. The spectroscopy setup covers a spectral window between 120 and 5000 nm, and the microwave interferometer can measure electron densities up to 1.5 × 10^20^ electrons/m^3^. The main design drivers and technological choices derived from the requirements are discussed in detail herein.

## 1. Introduction

Entry, descent and landing (EDL) is one of the most challenging mission phases for planetary exploration/Earth return spacecrafts, as one needs to ensure the safe and appropriate deceleration of a spacecraft until the soft landing at ground level.

Besides all the specific technological challenges related to the late stages of deceleration (descent and landing), with the definition of appropriate glide systems, parachutes, and landing systems (retropropulsion, inflatables or crushable structures), the atmospheric entry phase remains one of the most complex phases to be tackled. This flight phase occurs at extreme hypersonic speeds with strong deceleration and high heating rates, owing to the so-called *atmospheric entry plasmas*, which are created downstream of strong, detached shock waves typical of hypersonic flow regimes.

These shock waves convert the coherent energy of the flow into thermal agitation energy, impulsively heating it and triggering the internal excitation, dissociation and ionization of the flow species in severe nonequilibrium conditions, ultimately leading to the formation of the entry plasma. This nonequilibrium plasma also radiates significantly besides strongly convecting heat towards the colder spacecraft walls, triggering endothermic surface reactions, which, in general, lead to the ablation of the wall surfaces, which are protected by a thermal protection system (TPS). The precise knowledge of the physical–chemical properties for such plasmas is accordingly key to the efficient design of such TPS systems, among other aspects (aerodynamics, flight stability, blackout issues, etc.).

### Ground Testing

A shock tube is a facility designed to create a high temperature gas flow during a short time interval. It is comprised of a high pressure driver section and a low pressure driven section separated by a diaphragm. At a pre-determined pressure, the diaphragm ruptures, and the pressure discontinuity creates a shock wave moving towards the low pressure side. The shock wave will then excite the driven gas, forming a plasma. A simple single-stage shock tube typically cannot generate shocks speeds in excess of 10 km/s, typical of superorbital entries. To overcome this limitation, a double stage shock tube can be deployed, wherein an intermediate section between the driver and the test section is present, acting as a compression/acceleration tube.

**Figure 1:**
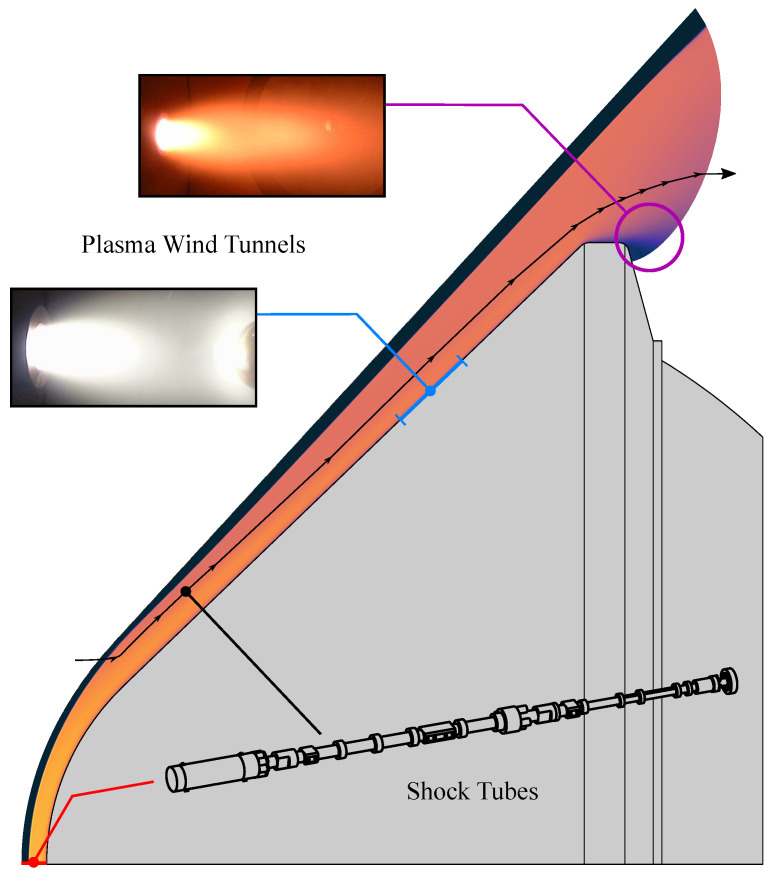
Schematic plot outlining the applicability range of ground test facilities for reproducing the different regions of an atmospheric entry flow. Shock tubes directly reproduce the stagnation streamline region where the heat fluxes are typically peaked (red dot), or other flow streamlines in the forebody (black dot and streamline). Free plasma plumes from wind tunnels are tailored for reproducing expansion regions (large violet circle), or surface ablation if an obstacle is placed inside the plume (blue dot and line). The plasma plume images (with and without obstacles) are taken from the SR5 arcjet plasma wind tunnel [20]. The simulated flowfield is adapted from a previous work reproducing the 1995 Jupiter entry by the Galileo probe [21].

Shock tubes are the most faithful facilities for adequately reproducing post-shock conditions for such flows, either directly reproducing the stagnation line flow (behind a normal shock), or indirectly reproducing the other forebody flow regions (behind an oblique shock) by means of straightforward correlations [1]. Such impulsive facilities are complementary with other steady-state plasma wind tunnels, which can, in turn, reproduce the conditions near the spacecraft walls, or afterbody expansions (note that shock tubes may also be deployed as impulsive wind-tunnel facilities, see Ref. [2] for more details). These facilities may also reproduce such flow conditions more approximately, owing to their different plasma excitation mechanism (electromagnetic field), whereas the excitation mechanism in a shock tube is the exact same one as that in flight conditions (shock wave). Figure 1 schematically shows the range of applicability for the different facilities. In short, shock tubes are (among others) key facilities in the planetary entry research ecosystem of space-faring nations.

Akin to the other world’s space agencies, the European Space Agency (ESA) supports fundamental and applied research on atmospheric entry plasmas to advance European planetary exploration endeavors. The earliest concerted studies on atmospheric entry flows at European level were carried out in the scope of the HERMES space shuttle development program [3], where two shock tube facilities were developed to support this endeavor: the HEG shock tube at Göttingen, which acted as an aerodynamic shock tube facility [4], and the TCM2 shock tube at Marseilles, which was devoted to fundamental studies on kinetic and radiative shock-induced processes [5]. The latter could only reach velocities of about 8–9 km/s, which was enough for reproducing a return from Earth’s orbit, the Huygens mission (which successfully entered Titan at a velocity of 5.15 km/s in 2004) [6,7,8] or Mars exploration missions [9,10,11], but not enough for recreating an Earth superorbital entry (11–12 km/s) [12]. However, the renewed ambitions at the beginning of the century, namely the Mars Sample Return mission, required a higher performance facility. In consequence, a competitive tender for the development of a novel facility was launched by ESA in 2009. This competition was won by an international consortium led by the Institute for Plasmas and Nuclear Fusion (IPFN), a research unit of Instituto Superior Técnico (IST) [13,14]. The European Shock Tube for High-Enthalpy Research (ESTHER) was developed in the scope of this contract and is in its final commissioning phase [15].

Such a higher performance shock tube facility requires state-of-the-art instrumentation. Atmospheric entry plasmas are very energetic, and thus radiate very strongly in a broad range of wavelengths. Spectroscopy is therefore a key diagnostic for probing such plasmas, also taking into account that these diagnostics are non-intrusive in nature, hence not disturbing the flow. Measurements of time-dependent emission/absorption in specific wavelength ranges allow probing for specific atomic and molecular quantum transitions, and may indirectly provide information on the time evolution of species concentrations (relative or absolute depending on the setup calibration), as well as flow and species temperatures, providing indication of departures from Boltzmann equilibrium. A direct measurement of the overall time-dependent radiative fluxes (in W cm^−2^) emitted by the shock wave factors in the TPS design, which needs to withstand the overall convective and radiative fluxes of the flow during the whole entry phase. Measurements carried out in the past were mostly restricted to optical emission spectroscopy from the near-UV to near-IR range, which encompassed most of the spectral features of interest in this velocity region [16,17,18]. However, at higher velocities, the more prominent radiative features move towards the ultraviolet-vacuum ultraviolet (UV-VUV) region, while on the other side, Mars entries also have peculiar radiative heating characteristics with a prominence in the mid-wave infrared (MWIR) [19]. Accordingly, two companion contracts were awarded to the consortium for the development of additional optical spectroscopy setups, one in the UV-VUV and another in the MWIR region.

Knowledge on the electronic densities of such plasmas is also a key parameter, as it drives the excitation of radiative states of the flowing species, via electron-impact excitation reactions. Information of heavy species excitation states is also important for the design of TPS [22], as heavy species will heat the wall through convective and radiative heat transfers, possibly endangering the spacecraft if such fluxes exceed the engineering limits [23]. The electron density may be inferred either through spectroscopic methods (Stark broadening techniques [24,25]), or through microwave interferometry techniques. Although these are, to date, not as developed as the formerly discussed optical spectroscopy diagnostics, several proofs of concept for such diagnostics have been demonstrated, and one may expect such techniques to be more vigorously deployed in the near future [26].

## 2. Materials and Methods

This section discusses the operational requirements of shock facilities in terms of shock speeds and ambient pressure, followed by the general requirements for optical spectroscopy measurements. Then, it discusses existing shock tube facilities for atmospheric entry studies, including our own and its expected performance. It then concludes with a more detailed discussion on the requirements for time-dependent optical spectroscopy and microwave interferometry for radiation and electron density measurements, respectively.

### 2.1. General Specifications and Requirements

**Figure 2:**
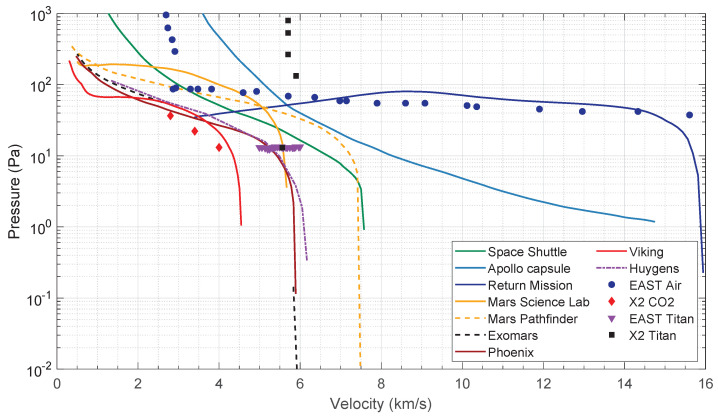
Entry conditions for different spacecrafts. Space Shuttle, Apollo and Return mission in Earth (N${}_{2}$-O${}_{2}$); Mars Science Lab, Mars Pathfinder, ExoMars, Phoenix and Viking in Mars (N${}_{2}$-CO${}_{2}$); and Huygens in Titan (N${}_{2}$-CH${}_{4}$). Adapted from [29].

Entry conditions are determined by the spacecraft’s orbit into the planet gravitational field. These are then reproduced in key points (typically the peak heating and peak dynamic pressure points), which are obtained through semi-empirical expressions [27,28] on adequate ground test facilities, such as ESTHER. Figure 2 shows the spacecrafts entry conditions in different atmospheres and the corresponding experimental points for different shock tube facilities.

Once the key trajectory points are known, one may estimate the post-shock temperatures that are reached using simple thermodynamic correlations. Namely, the total stagnation enthalpy $h_{stag}$ of a shock wave system may be expressed as the sum of the internal enthalpy *h*, and a kinetic term $v^{2}/2$ due to the flow velocity [30]. The stagnation enthalpy of the system pre- $h^{0}$ and post-shock $h^{1}$ can be assumed to be conserved, yielding Equations (1) and (2):

(1) $$h_{stag}=h+\frac{v^{2}}{2}\;=const.$$

(2) $$h_{0}+\frac{v_{0}^{2}}{2}=h_{1}+\frac{v_{1}^{2}}{2}$$

Prior to the arrival of the shock wave, the gas is in chemical equilibrium at a low temperature, around 300 K for a shock tube experiment, where the kinetic energy term is dominant on the left-hand side of Equation (2) $v_{0}^{2}/2\gg h_{0}$. After the shock, most of the kinetic energy is converted into thermal energy, which both excites the internal energy modes and heats up the gas. Thus, on the right-hand side of Equation (2), the internal energy term is dominant over the kinetic one $h_{1}\gg v_{1}^{2}/2$. These approximations yield Equation (3), with $c_{p}$ as the gas specific heat capacity at constant pressure:

(3) $$\frac{v_{0}^{2}}{2}\approx h_{1}=c_{p}T_{eq}\;.$$

The gas total specific heat capacity can be split into two contributions, a constant frozen-gas term $c_{p_{f}}$ (for an ideal gas), and a contribution from the internal degrees of freedom:

(4) $$c_{p}=c_{p_{f}}+\sum_{i}h_{i}{\left(\frac{\partial c_{i}}{\partial T}\right)}_{p}\;.$$

Inserting Equation (4) into (2), and solving for *T*, we obtain Equation (5a) and both its upper and lower temperature limits, $T_{max}$ (5b) and $T_{eq}$ (5c), respectively.

(5a) $$\begin{array}{cc} T & =\frac{h}{c_{p_{f}}+\sum_{i}h_{i}{\left(\frac{\partial c_{i}}{\partial T}\right)}_{p}} \end{array}$$

(5b) $$\begin{array}{cc} T_{max} & =\sqrt{\frac{v_{0}^{2}}{2c_{p_{f}}}} \end{array}$$

(5c) $$\begin{array}{cc} T_{eq} & =\frac{v_{0}^{2}}{2c_{p}\left(T_{eq}\right)}=\frac{h}{c_{p_{f}}+\sum_{i}h_{i}{\left(\frac{\partial c_{i}}{\partial T_{eq}}\right)}_{p}} \end{array}$$

**Figure 3:**
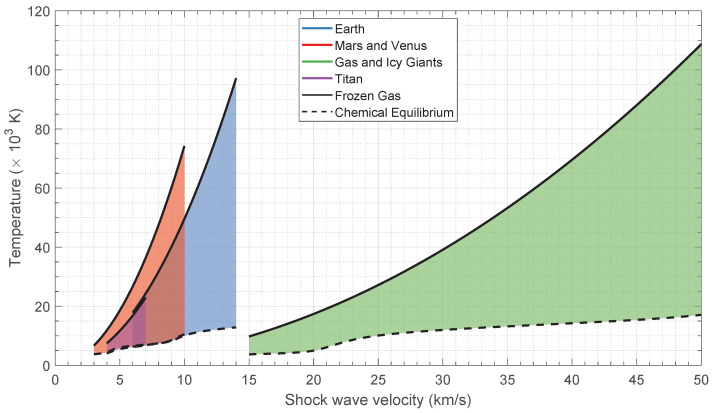
Temperature range for different entry conditions and atmospheres. Upper and lower limits correspond to a calorifically perfect and chemical equilibrium gas, respectively.

The expected post-shock temperature range of the gas is shown in Figure 3. The upper limit (full line) is the so-called frozen limit, where all the chemical reactions are ignored, and thus $c_{p}=c_{p_{f}}$; the lower limit (dashed line) is the final temperature after chemical equilibrium is reached. Chemical equilibrium temperatures have been computed using our in-house aerothermodynamics code SPARK.

**Figure 4:**
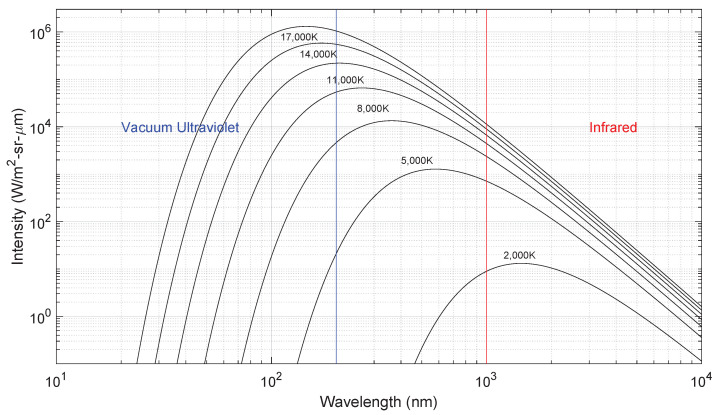
Planck blackbody emission wavelength distribution for various temperatures.

Figure 4 depicts the radiation wavelength distribution of a Planck blackbody at different temperatures. As the temperature increases, so does the emitted radiation power with $T^{4}$, in line with the law of Stefan–Boltzmann. In addition to this, the peak wavelength of the emitted radiation $\lambda_{peak}$ moves to the shorter wavelengths following Wien’s law $\lambda_{peak}\propto 1/T$ [31].

**Figure 5:**
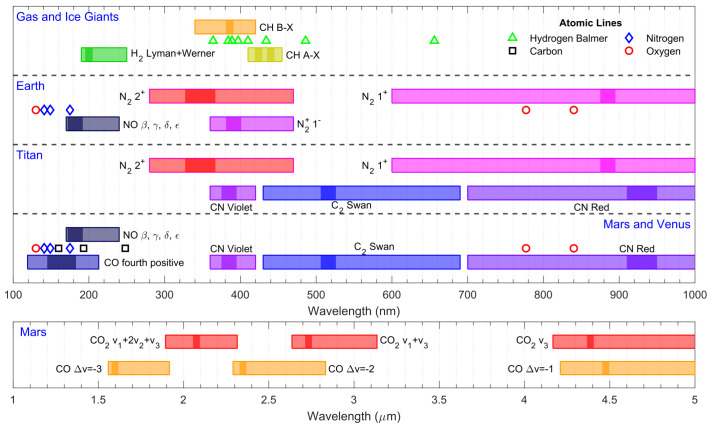
Emission region of different gas species in typical entry conditions for different planets. Observed atomic lines are explicitly presented for their measured wavelengths. Typically, molecular bands have strong bandhead peaks, with more diffuse tails in the neighbouring regions. To provide an estimate of the spectral regions where these bands may be emitting, we ran the in-house spectral line-by-line code SPARK [39,40] with a representative temperature of T=5000 K. We represent the bandheads with a stronger color, and the tails with the same faded-out color. The band heads/tail intensity ratio is taken as a factor of 100.

Typically, atmospheric entries occur at a gas pressure low enough that the emitted radiation cannot be assumed to be in equilibrium and treated as a blackbody, yet this assumption provides an upper limit for the emitted radiation over the whole spectral range. In general, the plasma is optically thin, and the discrete radiation spectrum is in strong non-Boltzmann equilibrium. The dominant spectral features will depend on the gas chemical composition and pressure, as well as the temperature (derived from the shock velocity). Entries faster than 7 km/s will usually ionize the gas and thus emit radiation in the ultraviolet region. Slower entries can only excite the molecular internal vibration levels, which radiate in the infrared region. Figure 5 shows the most important emission regions for typical planetary entries, which is based on experimental data from previous shock tube campaigns by different teams all around the world. Data for Earth, Mars and Venus radiative transitions were taken from [17,32], Bose et al. [33], and Cruden et al. [34], respectively. Data for Jupiter were adapted from the work of Cruden and Bogdanoff in [35]. Neptune data were taken from [36,37]. Titan data were reported by Magin et al. [38].

As expected, the radiative features for these different classes of entry flows are quite rich, with a great deal of measured atomic transitions in the VUV region (120–200 nm), the Balmer series of H in the visible, and the O atomic lines in the near-IR region (at 777 nm and at 849 nm, respectively). Molecular radiation features are also very rich, with many emitting systems from C_2_, CH, CN and N_2_ all over the visible range, and H_2_, CO and NO emitting in the VUV range. For the IR, one typically observes emission from the different rovibrational bands of CO and CO_2_ from 1.5 μm to over 5 μm. These spectral features may now guide us into selecting the appropriate measurement setup.

### 2.2. Operational Shock Tube Facilities for Fast Atmospheric Entries

Reynier et al. provide a detailed review on hypersonic facilities in [41,42]. Currently, the only ground test facilities capable of achieving superorbital velocities (above 10 km/s) are Ames Electric Arc Shock Tube (EAST—Moffett Field, CA, USA); X2 and X3 expansion tubes (Brisbane, Queensland, Australia); CUBRC LENS XX expansion tube (Buffalo, NY, USA); Hyper Velocity Shock Tube (HVST—Tokyo, Japan); TsAGI ADST shock tube (Moscow, Russia); and the T6 Stalker tunnel (Oxford, UK). Typically, shock tubes use emission spectroscopy to determine the chemical composition of the gas behind the shock wave in its non-equilibrium state. Chemical species concentrations may be determined via absorption spectroscopy. The electron density may be estimated via an indirect method, such as the Stark broadening of some emission lines. Nonetheless, some facilities use microwave interferometry to directly measure the electron density behind a shock wave. Electrostatic (Langmuir) probes are simple diagnostics used for electron density and temperature measurements [43]. However, these are intrusive diagnostics which perturb the flow in the diagnostics region and are damaged by it. Therefore, these drawbacks preclude its use in a shock tube.

EAST [24,44,45] at NASA Ames is equipped with time-of-arrival sensors to have a high-resolution velocity measurement. A long slot optical window is present for shock imaging via spectroscopic instrumentation. A total of four different sets of optics, each with its own spectrometer, perform the imaging at the same axial location. The spectrometers are selected as a function of the region of interest of the electromagnetic spectrum, which itself depends on the shock wave velocity, test gas pressure, and chemical composition. The regions are generally classified as vacuum ultraviolet (120–200 nm), ultraviolet/visible (200–500 nm), visible/near infrared (500–900 nm), near infrared (900–1600 nm) and mid wave infrared (1600–5500 nm). The VUV spectroscopy equipment must operate under vacuum conditions to prevent the ultraviolet radiation to be absorbed along its optical path, and the optical windows must be made of MgF_2_ or LiF to minimize absorption.

The University of Queensland hosts three expansion tube facilities, named X1, X2 and X3. The latter two facilities are capable of reaching superorbital velocities and produce VUV and UV radiation. The X2 expansion tube spectroscopy system [46,47,48] consists of a normal incidence spectrometer and an intensified charge coupled device (iCCD) with a camera of enhanced sensitivity in the VUV spectral range. The system has a theoretical resolution of 0.06 nm/pixel, and a range of 60 nm. The UV spectral region can also be observed using the same setup. An additional visible/NIR spectroscopic system is present, with a wavelength range of 695 to 880 nm and 0.55 nm/pixel resolution. The flow is also monitored with a high-speed camera. Besides the emission spectroscopy diagnostics, X2 has Nd:YAG (355 and 532 nm) interferometry instrumentation equipment capable of measuring density, ionization levels, species concentrations and temperatures [48].

The Japanese HVST [49] located in JAXA’s Chofu Aerospace Centre is a free-piston-driven shock tube which operates in both shock and expansion tube modes. A He-Ne laser Schlieren setup is used to detect the shock front and measure the shock wave velocity. Three spectrometers covering the VUV to NIR spectral region are coupled to two CCD arrays [50,51,52,53] for radiation emission spectroscopy.

The LENS XX is a hypersonic expansion tube in Buffalo, N.Y., equipped with an emission spectroscopy system [54] in the UV to visible with two gratings (1200 and 150 g/mm) and an iCCD camera. The calibration is performed with a deuterium lamp.

T6 [55] is a Stalker shock tunnel located at the University of Oxford, which may reach velocities up to 18 km/s for light test gases (H_2_-He). The emission spectroscopy setup [56] is based on the X2 expansion tube. A series of UV-enhanced aluminum mirrors focus the light into a spectrograph. A 550 nm longpass filter may be applied for measurements in the red and NIR region of the spectrum. The setup can operate in the 350–850 nm range using either gratings of 150 or 1200 g/mm.

#### 2.2.1. The European Shock Tube for High Enthalpy Research

**Figure 6:**
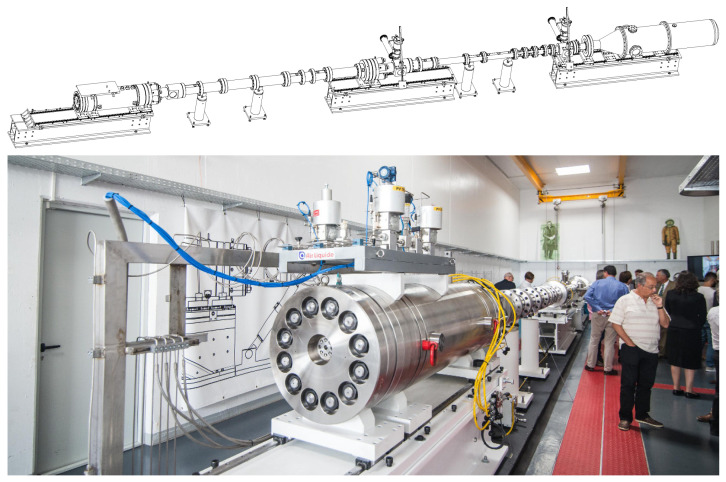
European Shock Tube for High-Enthalpy Research (ESTHER). Driver section to the left, test section on the right side. Shock wave moves from left to right.

ESTHER is expected to reach shock wave velocities in the range of 6 to 14 km/s in air or above 18 km/s in (H_2_-He mixtures [29,57]. Figure 6 depicts a schematic and a photograph overview of ESTHER. The facility is comprised of four sections, separated from each other by diaphragms. These are the combustion chamber driver, the compression tube, the shock tube (also called test section) and the dump tank. A small-scale combustion driver was previously tested in order to de-risk the development of the full-scale driver [14]. Whereas the qualification tests of ESTHER are ongoing [15], the tests of the combustion driver were successfully completed. The driver of ESTHER is a 47-liter cylindrical 200 mm internal diameter combustion chamber, capable of handling He:H_2_:O_2_ or N_2_:H_2_:O_2_ mixtures with filling pressures up to 100 bar and post-combustion deflagration pressures of 660 bar. The combustion chamber and its equipment are designed to operate in deflagration (subsonic combustion) mode. Nonetheless, the driver can withstand the detonations (supersonic combustion), which may occasionally occur creating transient pressures up to 1.8 kbar. The ignition of the mixture is attained using a high power Nd:YAG laser [58,59], which fires a 5 ns pulse into the chamber. The compression tube, with an internal diameter of 130 mm, is connected to the driver via a diaphragm designed to open at a predefined pressure. Once filled with helium at pressures between 0.01 and 1 bar, the shock wave moving along the compression tube can reach pressures of up to 70 bar. A second diaphragm divides the compression and the shock tube sections. The shock tube, with a 80 mm internal diameter, is filled with the test gas mixture at pressures between 10 and 100 Pa (0.1 to 1 mbar). The shock wave reaches velocities exceeding 10 km/s in the shock tube, leading to transient pressures reaching up to 20 bar. Pressure sensors and optical detectors are positioned along the shock tube to measure the shock wave velocity and trigger the time-dependent spectroscopic measurements at the test section. Lastly, a 1000 L dump tank, separated from the shock tube by a third diaphragm, recovers the gas flowing in the wake of the shock wave. Following each shot, the liquid phase is drained off, and the remaining contaminated gas mixture is evacuated by the vacuum pumps located in the shock tube section. The tube is then opened for cleaning and diaphragm replacement.

The facility is equipped with 20 ports at 6 different positions along the tube’s axial direction, 4 in the compression tube section and 16 in the shock tube section (8 in the test section). There are two measurement stations at the test section, each with four ports located circumferentially, which allow for multiple diagnostics and measurements at the same axial position. These optical ports have a 10 mm diameter cylindrical shape.

#### 2.2.2. ESTHER Performance Map

The ESTHER performance map was predicted using the STAGG code (Shock Tubes and Gas Guns), developed by Fluid Gravity Engineering Ltd. The code solves the set of shock tube equations given the design variables (cross-section areas, length, single/two-stage geometry) and numerical inputs of the chemical mixtures. The numerical model is based on the works of Alpher and White [60], Walenta [61] and Mirels [62]. The gas is assumed to be isentropic and inviscid [63] to compute the shock speed and the pressure along the tube. Further details of this development can be found in [29]. The code was first calibrated using the VUT-1 test data from ESA’s CO_2_ validation campaign [64]. The STAGG simulations were later re-run following the driver qualification campaign, once the driver combustion performance was assessed [57] in order to have a more realistic performance envelope of the facility.

**Figure 7:**
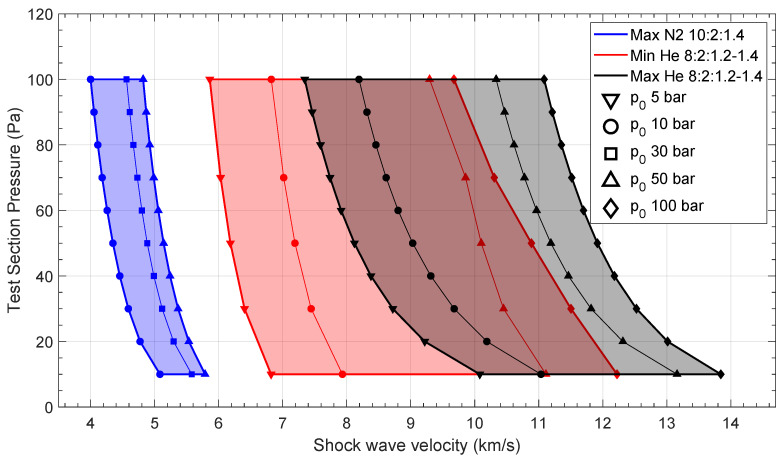
Expected ESTHER performance envelope (test gas pressure vs. shock wave velocity) for air (N${}_{2}$-O${}_{2}$) from STAGG code.

STAGG simulations were performed in two different modes, optimization and non-optimization. In the first case, the compression tube pressure is adjusted to maximize shock wave velocity at the test section, and in the latter, all input parameters are fixed and the code solves the equations to compute the shock wave pressure, temperature and velocity [63]. Post-combustion temperatures and pressures were obtained during the driver qualification campaign for different gas mixtures and filling pressures [65]. During this campaign, the nominal operational mixtures were tuned to He:H_2_:O_2_ 8:2:1.2–1.4 for the high-velocity experiments (>7 km/s) and N_2_:H_2_:O_2_ 8:2:1.4 for the low-velocity experiments (<6 km/s). To generate the performance map, STAGG ran three sets of simulations: Helium driver optimization; Nitrogen driver optimization; and Helium driver non-optimization. These correspond to high (>7 km/s), low (<6 km/s) and medium (6–7 km/s) velocity regions. The input parameters for the different sets of simulations are shown in Table 1. Maximum performance is found when STAGG runs with optimized conditions; however, running it in non-optimized (de-tuned) conditions is also useful to achieve lower shock velocities, extending the operational range. Figure 7 shows ESTHER performance simulation for Earth’s (N_2_-O_2_) atmosphere. The envelope is drawn using the lowest and highest velocity points of each simulation group.

### 2.3. Optical Spectroscopy Specifications and Requirements

Section 2.1 provides us with the operational conditions, pre-shock pressure and target speed, for our setup, and more specifically, Figure 5 provides us with the specific wavelengths of interest for the different gas mixtures considered in the testing. However, the wavelength alone is not sufficient for the specification of the equipment, as one needs to consider as well the shock speeds. These will define the acquisition time, and in turn, the temporal/spatial resolution of the system. The design of the trigger system and of the fast spectroscopy electronics is also influenced by the shock wave speed; the total time should be about 20 μs at worst (for maximum operational speeds).

Another two important parameters are the spectroscopic resolution and the sensitivity of the system. In terms of the spectroscopic resolution, typical spectroscopic systems which work in monochromator mode try to target the best possible resolution, as they are typically deployed in steady-state experiments, where the wavelength can be slowly scanned and acquisitions (either via a photomultiplier or an intensified camera) can be tailored to be long enough so that a good signal-to-noise ratio is achieved. An example for such a setup is described in Ref. [66]. In the case of a shock tube facility, we are collecting light trailing a moving shock wave for a few μs in an experiment that is typically one or few hours in the making. Not only is a spectrograph setup (imaging the full spectral window in one sweep) mandatory, but one needs to ensure that the maximum amount of light is collected during the passage of the shock wave. Therefore, there is a need to ensure that the setup collects the maximum possible amount of light, as well as a need to tailor the spectral window. For the latter, higher spectral resolutions yield lower spectral windows and vice versa. The spectral resolution is determined by the gratings installed in the spectrograph, which can be exchanged. The separation of different wavelengths is called the angular dispersion D and can be computed as the derivative of the reflection angle $\theta_{m}$ with respect to the wavelength λ:

(6) $$D=\frac{d\theta_{m}}{d\lambda }=\frac{k}{a\cos\theta_{m}}\;,$$

where *a* is the groove distance in the grating (inverse of the groove density). The reflection angles in Equation (6) can be calculated by

(7) $$a(\sin\theta_{i}+\sin\theta_{m})=k\lambda \;,$$

where *k* is the order of diffraction (1, 2, …). For a set of *a* and $\theta_{i,m}$, multiple λ satisfy Equation (7). The lowest-order solution, k=1, corresponds to the longer wavelength $\lambda_{1}$, with higher order solutions corresponding to $\lambda_{k}=\lambda_{1}/k$. The dispersion of the wavelength at the spectrograph focal plane with focal distance *f* is computed through

(8) $$\frac{dy}{d\lambda }=fD=\frac{fk}{a\cos\theta_{m}}\;.$$

A first-order Littrow blaze can be applied to the diffraction grating. It increases the intensity of the refraction order and wavelength of interest by curving the grating surface to direct the light at a preferred angle. These governing equations are useful for defining which gratings will be best suited in terms of apparatus function and spectral window.

Broadly speaking, three different spectral regions (each with its own peculiar characteristics) are identified:

1. The ultraviolet and vacuum ultraviolet regions where most of the radiation for high-speed entries should be emitted (see Figure 4). This region is bounded roughly between 120 nm (below which most windows become opaque) and 300 nm (where the visible region begins).
2. The near-UV to near-IR region in the 300–850 nm range, colloquially referred as the visible range, where many atomic and molecular systems are emissive for moderate entry speeds (see Figure 5).
3. The near-IR to mid-IR region (roughly in the 1–5 μm range) where rovibrational transitions between heteronuclear molecules, such as CO_2_, CO, and NO, are strongly emissive. Radiation is observed in this spectral range for low-speed entries in planetary atmospheres with such gases in their composition (mostly Mars, for which the large impact of IR CO_2_ radiation was recently assessed [19,67]).

Each of these three spectral regions requires its own setup. This means selecting an adequate spectrograph/camera combo. In terms of spectrographs and beyond the selection of appropriate gratings, one needs to account for the absorption of room air, which encompasses all the spectral ranges below roughly 200 nm, and specific bands in the IR (owing to the trace amounts of CO_2_ and water vapor). This means that a VUV setup needs to be held in vacuum, and an IR setup typically needs to be flushed in an inert non-IR absorbing gas, such as Nitrogen. The camera itself needs to be sensitive to the spectral range of interest. Intensified high-speed cameras (iCCD) encompass all the spectral ranges of interest of this work, whereas streak cameras are limited to the UV-VUV and visible ranges. Streak cameras have an advantage over iCCD cameras in the sense that the temporal variations of light intensity at an imaged point are translated to variation in image brightness along the streak direction. In contrast, iCCD cameras sample a line of points where the shock wave evolves over a given acquisition time, hence translating spatial points into time through the shock wave speed. These cameras are subject to motion blur, whereas streak cameras are not. Nonetheless, the deployment of the latter for higher speeds is more arduous. Whereas IR is the only spectral region where streak cameras do not exist, the shock waves are slower and therefore the issue of motion blur is less critical.

**Figure 8:**
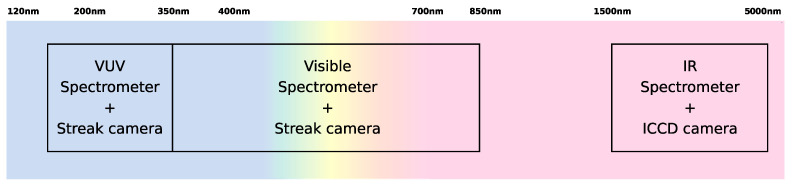
ESTHER instrumentation spectral range coverage.

The optimal setup selection is therefore schematized in Figure 8. The detailed specifications and requirements for these equipment, as defined by ESA, are presented in Table 2 and can now be discussed in detail. Note that no specifications and requirements have been defined in the visible region, as the optical setup from the previous TCM2 shock tube is reused.

These specifications and requirements were defined taking into consideration the generic characteristics for spectroscopic setups in their spectral regions. These are understood to be adjustable for a shock tube configuration. In terms of spectral range, resolution and accuracy, most of the commercially available spectrograph equipment are compliant to the specified parameters. In turn, most of the streak and iCCD cameras are capable of achieving integration times lower than 1 μs. The specification of the signal-to-noise ratio will be more experimentally dependent on the amount of radiation emitted by the shocked flow, which then will drive the requirements to minimize stray light and general noise from the acquisition system.

With this said, the assembly and deployment of a setup for detecting short bursts of light typical of shock tube experiments entail a certain number of restrictions, which immediately narrow down the range of compliance to the specifications and requirements of Table 2. Among others, one needs to account for window transmissivity; spectral response, range and sensitivity of the streak/iCCD camera photocathode/CMOS sensor, respectively; bit-rate for the high-speed electronics of the cameras; and availability of fiber optics to connect the spectrograph to the shock tube optical windows. All these narrowed-down specifications and requirements are discussed in detail in Section 3 (taking into account the spectral region, either UV-VUV or IR), except for the window transmissivity, which is discussed here.

**Figure 9:**
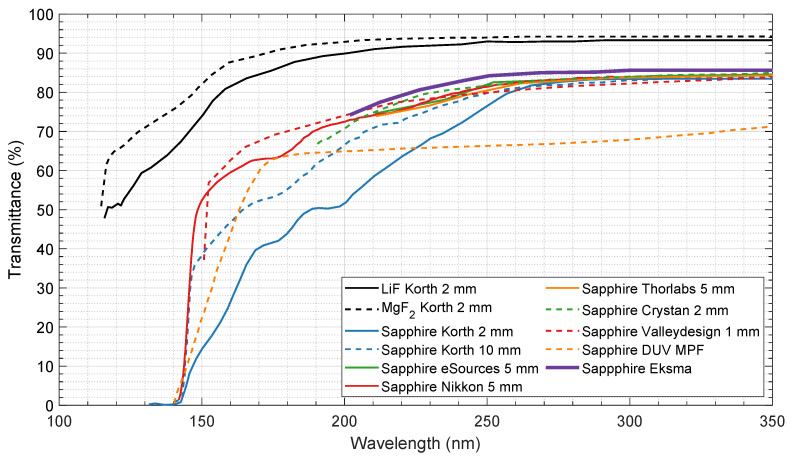
Optical transmissivity for common window materials, data adapted from [69,70,71,72,73,74,75,76].

VUV light is easily absorbed by gas molecules, and most optical materials, such as quartz or glass. This mandates the VUV system be held in vacuum to prevent the collected light from being absorbed by oxygen and nitrogen molecules. Using an optical fiber cable is also not possible as these have strong attenuation below 300 nm. Alongside, the optical window material should be as transparent as possible in the 100–300 nm range. Figure 9 and Table 3 show the optical transmissivity of different materials in the VUV region. Usually, VUV windows are made of Lithium Fluorite (LiF) or Magnesium Fluorite (MgF_2_); however, these materials cannot handle the force of the passing shock wave and will break after two or three experimental runs. For this reason, VUV-graded sapphire was the material chosen for the windows of the spectroscopy system. Regarding the NIR-MWIR spectral region, all the aforementioned optical windows materials are essentially transparent up to 4–5 μm, and there is the additional advantage that infrared optical fibers are available to “transport” the light signal from the shock tube to the spectrometer. These have low attenuation in the infrared region [68] and do not require vacuum like the VUV wavelengths.

### 2.4. Microwave Interferometery Specifications & Requirements

Interferometry is an adequate technique for electron density diagnostics in shock tubes since these lack any radial profile (in other words, the shock wave front moves as a “disk” over the tube). Other than a small test campaign carried out at the Moscow Institute of Physics and Technology [41,64,77,78], in the scope of an ESA contract, there are no further direct electron density measurements in hypersonic shock tube facilities using this technique to the authors knowledge. Most plasma interferometry measurements were performed in combustion shock tubes [79] or to measure ionization rates [80]. Interferometry is a common diagnostic in plasma plumes, namely Hall thrusters, because of its non-invasive nature. Examples of this diagnostic include measurements made in Xenon [81,82,83,84,85], and Hydrogen [86]. A recent review on the applicability of this technique for atmospheric entry applications is provided in [26].

The plasma cut-off frequency $f_{pe}$ is the minimum value at which an electromagnetic wave can propagate in a plasma. It relates to the critical electron density $n_{e,p}$ via Equation (9) [43], where $\varepsilon_{0}$, *e* and $m_{e}$ are the vacuum permittivity, the electron charge and mass, respectively:

(9) $$f_{pe}=\frac{\omega_{p}}{2\pi }=\frac{1}{2\pi }\sqrt{\frac{n_{e,p}e^{2}}{m_{e}\varepsilon_{0}}}\;.$$

The cut-off frequency is a key parameter to design any microwave diagnostic. In microwave reflectometry, commonly used in fusion reactors [43,87], the electron density profile is diagnosed using a radar-like technique with microwaves. A probing signal is emitted to the plasma, where it propagates until a layer with $n_{e}$ equal to the critical value $n_{e,p}$ is found, and is then reflected. The phase difference of the two signals relates to the time of flight of the probe signal and to the plasma refraction index. The latter is associated with the plasma density via the Altar–Appleton equation [88]. Microwave interferometry uses a similar principle to reflectometry; however, the probing signal must completely transverse the plasma. The phase gained by the signal compared to the reference will relate to the average electron density of the plasma. To create a spatial plasma profile, the signal must be de-convoluted via Abel-inversion (“onion-peel”) techniques. Both techniques can work monostatically with one antenna for wave emission and reception, or bistatically with one antenna dedicated for emission and another for reception. More details on the specifics for this technique may be found in Ref. [89].

The functional requirements of an interferometer mandate that it should be compact and self-sufficient so it can be assembled and tested in different facilities with ease. Namely, its antennas, emitting and receiving, must be compatible with ESTHER optical plug windows (diameter 10 mm). Its working frequency (*f*) must be sufficiently high to traverse the plasma without reflecting back, and gain a phase delay [89] significantly large to be detected. The phase shift is given by Equation (10), where *D* is the plasma thickness, and $\omega_{p}$ and ω are the plasma oscillation and probing angular wave frequencies, respectively:

(10) $$\Delta \phi =\frac{2\pi }{\lambda }\int_{0}^{D}\left(1-\mu \right)dl=\frac{2\pi }{\lambda }\int_{0}^{D}\left(1-\sqrt{1-\omega_{p}^{2}/\omega^{2}}\right)dl\;.$$

Using Equation (9), λ=c/f, and ($\omega \gg \omega_{p}$), Equation (10) can be simplified into

(11) $$\Delta \phi ≃\frac{e^{2}}{4\pi cm_{e}\varepsilon_{0}f}\int_{0}^{D}n_{e}dl=\frac{e^{2}}{4\pi cm_{e}\varepsilon_{0}}\frac{\bar{n_{e}}D}{f}\;,$$

where $\bar{n_{e}}$ is the average electron density over the plasma path. This integrated value is a very good approximation to shock tube measurements, as the plasma can be approximated to a disk whose properties change only in the longitudinal direction. Optimally, the working frequency should be high enough to transverse the plasma and guarantee D/λ>3. However, if it is too high (small λ), the phase gained may be too small and too difficult to measure. Alongside working as a interferometer, the base equipment base should be convertible to a reflectometer to be mounted on a small spacecraft.

**Figure 10:**
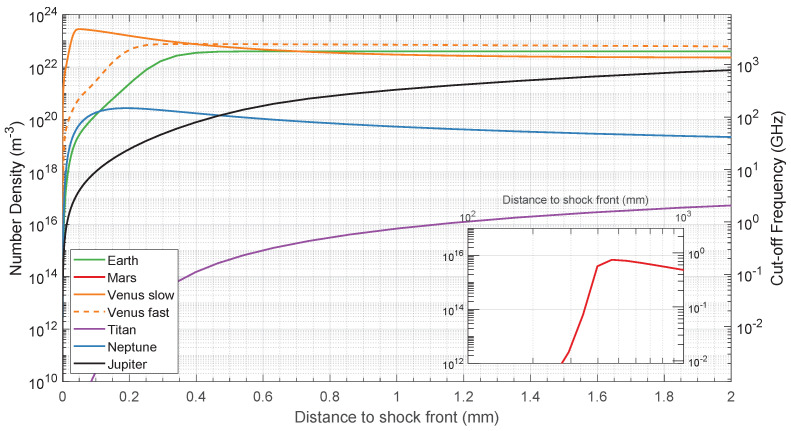
Electron density in shock tube for typical entry conditions in different atmospheres.

Since not much experimental data are available for direct electron density measurements, a set of CFD (Computational Fluid Dynamics) simulations was performed to estimate the required range for the diagnostics equipment. Shock wave conditions were estimated through the chemical reactive CFD code SPARK (Software Package for Aerothermodynamics, Radiation and Kinetics) [90]. The code is capable of computing the chemical composition behind the shock wave and its respective electron density and emission radiation. A total of six simulation runs were carried out in 1D (post-shock relaxation) conditions. Table 4 shows the CFD simulations initial conditions and references for the chemical–kinetic reactions schemes, as well as for the chosen velocity and pressure conditions. The representative cases are a sample return mission to Earth, and the ExoMars, Huygens and Galileo missions to Mars, Titan and Jupiter, respectively. Simulation conditions for Neptune and Venus were taken from trajectory calculations. The electron densities for these cases are depicted in Figure 10, where the electron density can reach values of $4\times 10^{23}$ electrons/m^3^ for the case of a Venusian entry. The typical electron density profile has a sharp rise right after the shock front, followed by a slower decay until chemical equilibrium is achieved.

As a side note, one needs to point out that Stark broadening measurements of the H-α and H-β lines provide an interesting technique for electron density analysis [24,25]. This may be considered a non-intrusive diagnostic to some extent, as a small percentage of the flow is replaced with Hydrogen (~0.1%). The Hydrogen atom α line at 656 nm and Balmer β line at 486 nm then typically become visible without significantly perturbing the overall spectrum. The observed lines may then be fitted to a Lorentzian curve with all other individual broadening contributions, van der Waals collisional, Doppler and instrument, accounted for. The FWHM (full width at half maximum) for the emission peak may then be compared to tabulated values, such as the ones found in [97], which give an estimation of the electron density. As a caveat, we note that the addition of (even small) quantities of H into the flow may affect wall desorption and slightly alter the flow properties; however, this effect should be relatively limited as shown by Cruden [24].

## 3. Results and Discussion

The specifications and requirements, as defined in the previous section, led to the final setup for ESTHER’s optical spectroscopy setup, which is now discussed in detail:

- A UV-VUV spectroscopy setup covering the 120–350 nm range;
- A visible spectroscopy setup covering the 350–850 nm range;
- A MWIR spectroscopy setup covering the 1–5 μm range.

Here, the spectral regions are bounded by the window transmissivity and grating efficiency of the UV-VUV setup, the streak camera input optics and photocathode sensitivity of the visible setup, and the optical fiber transmissivity of the MWIR setup. For each of these, appropriate blazed gratings are selected, which ideally maximize throughput on a specific wavelength of interest, while maintaining enough transmission efficiency over the whole spectral window. Lastly, finer/coarser gratings are selected to maximize spectral resolution/range over a single measurement, respectively.

In addition to the spectroscopy setups, an interferometer with a 2–18 GHz back end, and a 70.8–112.8 GHz front end was developed for the time-dependent measurement of electron densities. These four setups are discussed in detail in this section.

### 3.1. UV-VUV Spectroscopy Setup Design and Acceptance Testing

The selected equipment for UV-VUV spectroscopy setup is a McPherson Model 234/302 with an f/4.5 aperture and 200 mm focal length, coupled to a UV-VUV-customized Hamamatsu Streak Camera M10913-11, acceptance tested in November 2019. The use of a streak camera presents advantages over a simple iCCD camera. The most relevant is that the signal becomes time resolved since the streak camera creates a time discrimination of the received light. However, increasing the number of elements on the optical path reduces the signal-to-noise ratio. Streak cameras are based on the photoelectric effect, thus they do not work with low energy photons, namely in the infrared region. Therefore, any infrared spectroscopy instrumentation cannot make use of a streak camera.

**Figure 11:**
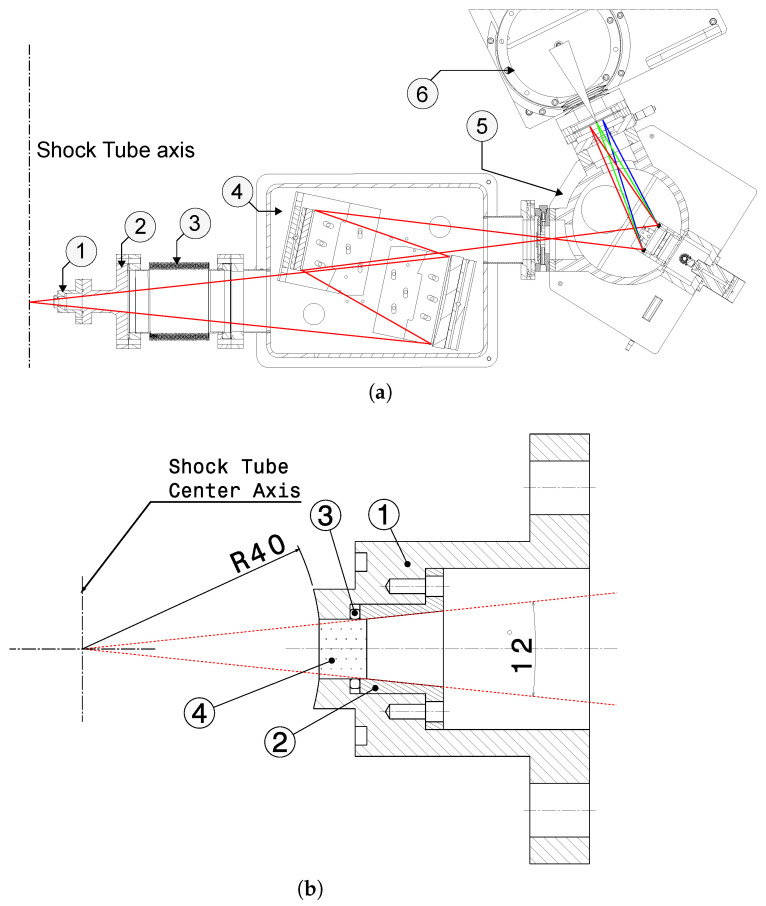
ESTHER UV-VUV spectroscopic setup design schematic. General design (**a**) and optical plug detail (**b**). In (**a**), 1—Shock tube plug; 2—Connection piece; 3—Adjustable Bellows; 4—Optics collection box; 5—Spectrograph with diffraction grating; 6—Connection to streak and CCD camera. In (**b**), 1—Plug; 2—Window tightener piece; 3—Viton O-ring; 4—Sapphire window. Red lines represent the optical path.

**Figure 12:**
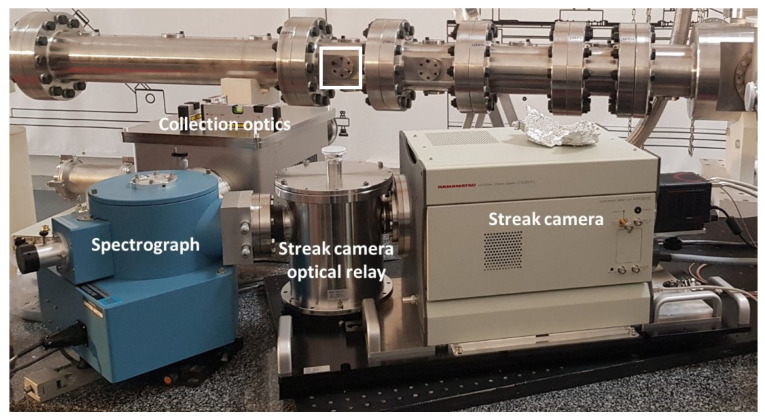
ESTHER UV-VUV spectroscopic setup during acceptance testing, ST8 optical port marked as a square in photo.

Figure 11 shows the overall assembly of UV-VUV spectroscopy system and a detailed view of the optical plug, which connects the test section to the UV-VUV spectrometer. The design maximizes the collected light via a 12° light cone throughout the optical path. An optical plug with a VUV graded sapphire window is held in place and vacuum sealed using one viton O-ring, and is connected to a collection optics box by flexible bellows (which allows connecting the shock tube, standing over a seismic slab, to the optics table standing in the experimental hall floor). The optics box is comprised of two mirrors, which collect and focus all the light cone into the spectrograph, hence maximizing the signal throughput. The spectrograph is in turn connected to the streak camera optical relay, which focuses the light into the photocathode that converts the photons into electrons, and deflects them using a saw-tooth signal. The streak camera is coupled to an iCCD camera (model ORCA-Flash4.0 C13440-20CU), which records the final time- and wavelength-dependent signal. Figure 12 depicts a photograph of the UV-VUV spectroscopy setup.

**Figure 13:**
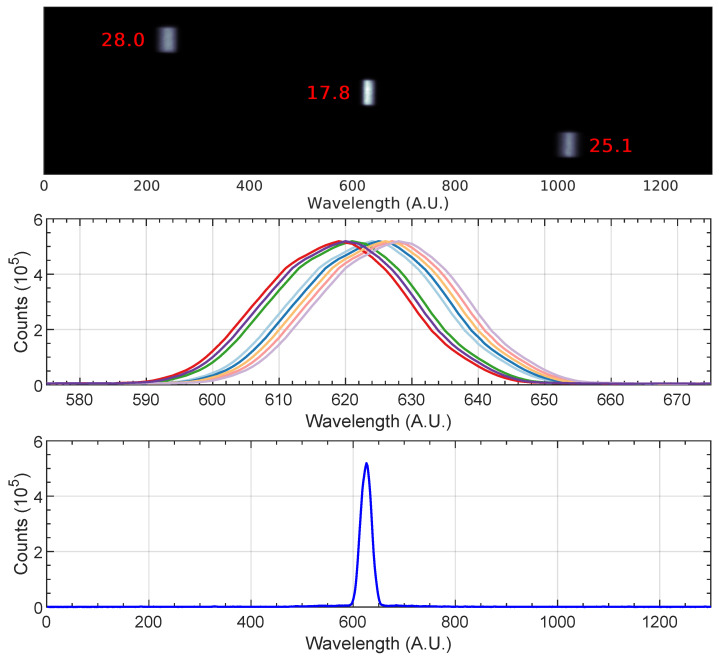
UV-VUV spectroscopic setup acceptance campaign results. Wavelength resolution in pixels for the 1200 g/mm grating (**top**); repeatability and accuracy (**middle**); and signal-to-noise ratio (**bottom**). Each curve in the middle figure represents a different run.

The spectrograph was equipped with two different diffraction gratings of 600 and 1200 g/mm blazed at 150 nm, ensuring an adequate throughput in the 120–350 nm spectral range. Validation tests for the setup were conducted with a mercury vapor lamp and a laser impulse generator. First, the equipment was calibrated with the 1200 g/mm grating using the Hg 253.65 nm line. The center pixel is located at number 628, with a total range of 782 pixels. Using the 1200 g/mm grating, a spectral range of 10 nm can be observed by the streak camera. The spectral dispersion is 10 nm/782 pixels = 0.0128 nm/pixel. The line FWHM at three different points of the photocathode was computed directly by the streak camera computer program to determine the wavelength resolution. Using the coarse grating, the center location was found to be pixel 618. The spectral range is now doubled to 20 nm with 803 pixels, thus giving a spectral dispersion of 20 nm/803 pixel = 0.0249 nm/pixel. The wavelength resolution values are presented in Table 5, and an example for the 1200 g/mm grating is depicted in Figure 13 (top).

The accuracy and reproducibility were tested by measuring the peak 10 times while sweeping the camera spectral center position. The average center position was found to be at 632.5 ± 4.5 pixels. Lastly, the signal-to-noise ratio was evaluated by the ratio between the peak intensity (513.79) to the noise standard deviation (0.15), yielding a signal-to-noise ratio 513.79/0.15 ≃ 3400. The test values for reproducibility and signal-to-noise are depicted in Figure 13 in the middle and bottom, respectively. The results from the UV-VUV acceptance campaign were in conformity with the requirements. However, the temporary unavailability of the vacuum pumps prevented testing the equipment with the deuteurium line at 110 nm or the mercury line at 237.83 nm. The streak camera sampling rate was tested with a picosecond laser. Table 6 presents the outline of the UV-VUV acceptance campaign.

### 3.2. Visible Spectroscopy Setup Description

**Figure 14:**
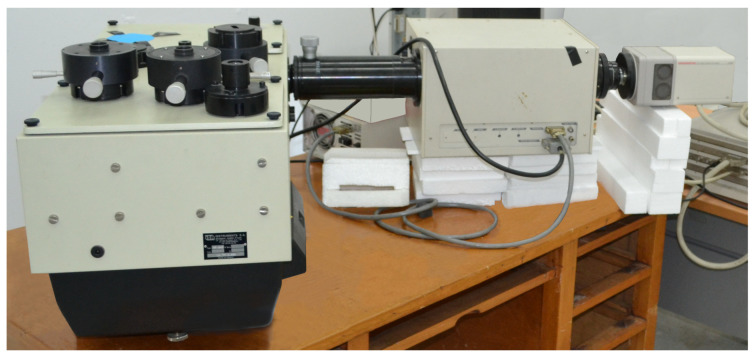
Visible range spectroscopy setup.

The visible spectroscopy setup used here was recovered from the previous TCM2 shock tube facility in Marseille, France, and is only be briefly outlined here (see Figure 14). The setup was used previously in a series of spectroscopic studies at the aforementioned facility [5,7,16,98]. It is comprised of a 0.64 m focal length Czerny–Turner Jobin–Yvon HR640 spectrograph f/5.2 using the following gratings: two low-resolution large-window 600 and 1200 g/mm gratings blazed at 500 nm, and two high-resolution narrow-window 2400 and 3000 g/mm gratings blazed at 330 nm, and at the 250–550 nm region, respectively. The spectrograph is coupled to a visible-range Hammamatsu streak camera comprised of a M1953 slow speed streak sweep unit and a universal temporal disperser C1587. The streak tube is the YD2369/N1643-01 with spectral range 200–850 nm and input optics model A1975 with spectral range 350–850 nm. A CCD camera (Hamamatsu C4880) is used to record the spectral-time images.

### 3.3. MWIR Spectroscopy Setup Design

**Figure 15:**
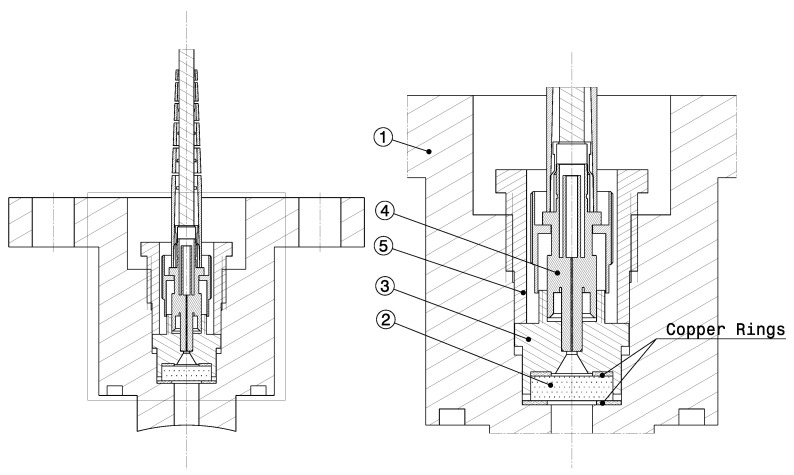
Optical plug for infrared spectroscopy diagnostics. 1—Plug; 2—Sapphire window; 3—Optical fiber connector; 4—Optical fiber cable; 5—Threaded screw tightening the window and optical fiber connector.

The infrared spectroscopy setup is currently in its late definition phase. The preselection of its main components is being carried out, namely, an optical fiber cable transparent in the MWIR, a spectrograph tailored for this spectral region, and a fast IR iCCD camera. The possibility for using an optical fiber cable connected to the shock tube allows for significantly more flexibility in the setup, compared to the UV-VUV system, which requires a bulky, vacuum-purged physical connection between the shock tube and the spectroscopy setup. Figure 15 depicts the optical plug scheme of the aforementioned setup. The optical fiber (4) is tightened to a part (3), which compresses the copper rings and seal the optical window (2), and it is connected on the other end to the spectrometer and the high-speed camera. This design is inspired by the shock detection system of the X2 facility at the University of Queensland, where the optical fiber is connected to a photodetector [99].

Table 7 shows the pre-selected equipment for the infrared spectroscopy setup. Optical fiber cables can either be single or multimode. The former has a higher peak transmittance but a narrow window, while the latter has a lower peak transmittance but broader transmission band. A large core-diameter fiber is also desirable to augment the collected light and provide an adequate signal-to-noise ratio. However, it may distort the signal over time due to possible differences in the travel distance between two photons. All the fiber patches are to be mounted with a ferrule connector on both the spectrograph and shock tube sides. Due to our broad spectral window, the chosen fiber must be a multimode one. For this case, indium fluorite core fibers have a slightly broader transmission band when compared to zirconium fluorite (ZrF${}_{4}$), despite the latter better transmissivity of the latter in the 2 to 3.6 μm region [68]. Both Thorlabs and Le Verre Fluoré propose fibers made of indium trifluoride (InF${}_{3}$), which presents good transmittance in the 1 to 5 μm region. The CIR fiber from Art Photonics has a well in the transmittance near 4 μm, and thus the overall signal-to-noise ratio would be lower than for the InF${}_{3}$ fibers. The selected optical fiber is thus a multimode indium fluorite (InF${}_{3}$) with a 200 μm core and 2 m of length.

Similarly to the UV-VUV and visible setups, an imaging spectrograph is needed. Table 8 presents popular equipment capable of working in the IR region. These are essentially spectrographs similar to the ones used in the visible range, except they have the possibility of being vacuumed or flushed with inert/dry gases, hence avoiding room air absorption from CO_2_/H_2_O traces. Longer focal length spectrographs yield larger wavelength separations; however, the spectral window narrows down accordingly. Additionally, wavelength resolution, accuracy, and window will naturally also depend on the selected grating.

**Figure 16:**
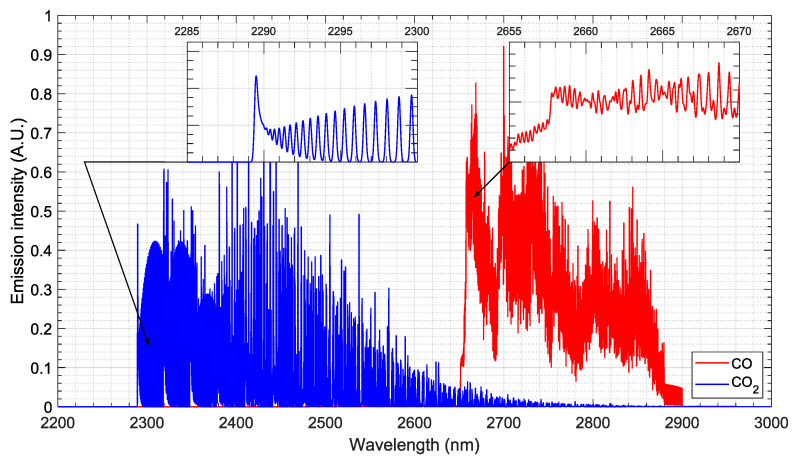
Synthetic spectrum for CO (blue) and CO${}_{2}$ (red) radiation in the IR. The spectrum is normalized for both radiative systems and assumes an equilibrium temperature of 3000 K and an apparatus function of 0.2 nm.

The selected imaging spectrograph is a McPherson 2035 equipped with two different diffraction gratings. A finer one with 300 g/mm is blazed at 2.5 μm for higher resolution, and a coarse one with 17.5 μm is blazed at 4.2 μm for a larger spectral window. The setup characteristics for coarse, broadband measurements are very close to the ones of the EAST shock tube at NASA Ames, which provided good-quality data for CO${}_{2}$ shocked flows in this spectral region for Venus and Mars atmospheric entries [100]. For the high-resolution grating, the specifications were taken considering an optimized spectral region where CO and CO${}_{2}$ bands may be observed with little interference. The 2–3 μm region is attractive for this purpose as shown by a spectral simulation for the equilibrium radiation of CO and CO${}_{2}$ at 3000 K equilibrium temperature, with an apparatus function of 0.2 nm, carried out with the SPARK Line-by-Line code [39,40]. Figure 16 shows the spectral features for these bands. CO rotational bands should be well defined and easy to probe in the 2.3–2.6 μm region, whereas CO${}_{2}$ bands would be prominent in the 2.65–2.9 μm region. The rotational features, allowing determining characteristic temperatures, are evident (see details in Figure 16).

Finally, a group of infrared cameras was also pre-selected for the setup. Table 9 lists the various cameras identified together with the corresponding specifications. A low minimum integration time is desired to avoid excessive smearing of the image in the iCCD since, as previously discussed in Section 2.3, a temporal discrimination of the infrared signal cannot be performed with a streak camera. A short integration time results in minimal smearing of the moving radiation signal; however, short integration times decrease the signal-to-noise ratio. Due to the fast-moving shock wave (up to 6 km/s), a 1 μs integration time is equivalent to a spatial smear of 6 mm in the worst-case scenario. Higher-resolution cameras have more pixels, allowing for a higher spectral resolution without sacrificing the width of the spectral window. Similarly, a higher temporal/spatial resolution can be achieved by increasing the pixel density. A characteristic that is not relevant for our experiment is the acquisition speed/full frame rate. The full frame rate of a fast iCCD camera is limited by the bandwidth of the camera electronics, not by the CCD sensor itself. Faster frame rates may be achieved at the cost of trimming pixels of the camera sensor, thus taking pictures with lower resolution. However, due to the nature of the shock tube experiments, only the first frame is relevant, as it captures the whole useful run time up to the contact wave, and therefore, this is not a concern for our application.

The spectral cameras of interest exhibit similar characteristics, with the cost driven primarily by the capability of the equipment electronics to achieve high-resolution, high-frequency frame rates. Since this is of no concern for our application, the final selection criteria will be likely driven by equipment cost.

### 3.4. Microwave Interferometry

**Figure 17:**
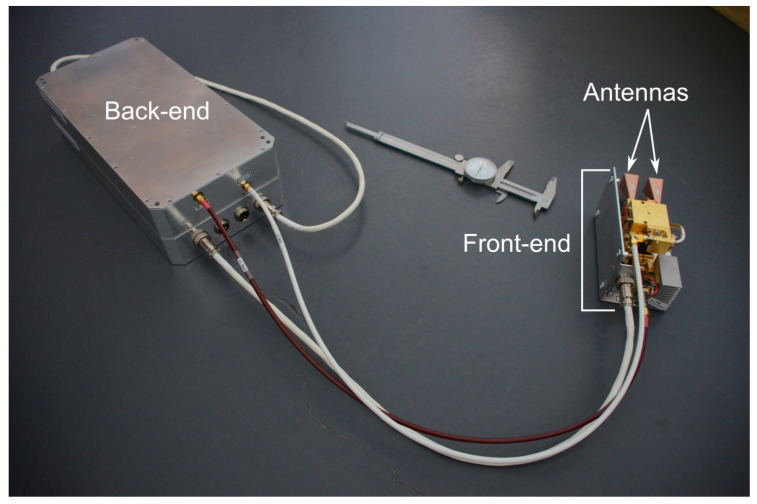
70.8–112.8 GHz interferometer.

As discussed in Section 2.4 and shown in Figure 10, the working frequency of a microwave diagnostic will depend on the electron densities of interest. These can fall into the microwave (3–30 GHz), the millimeter-wave (30–300 GHz), or the sub-millimeter-wave (>300 GHz) region. Accordingly, an interferometry system needs to be flexible to encompass several frequency-band segments and switch among them to scan a specific range, or alternatively, it may consist of a stack-up of simpler instruments running in parallel. This latter approach allows the development of very compact instruments and does not limit future extensions of the bands to cover. A bespoke equipment [26], depicted in Figure 17, was developed according to this design philosophy, which is composed of two sections: a back-end signal generator and a front-end frequency multiplier and antenna assembly.

The back end is a complete, digitally controlled, signal-swept generator operating at a convenient microwave frequency range, such as 8–12 GHz or 12–18 GHz (or 10–20 GHz). The fast-swept signal is generated inside the back end by ramping the control voltage of a VCO (voltage controlled oscillator). There are several VCOs available that cover a variety of frequency ranges, but none of them, without exception, exhibit a linear relation of control voltage versus frequency. Therefore, the proper sweep of a specific frequency range requires pre-distorted voltage ramps to obtain a linearized frequency sweep at the output. This is achieved by a digital arbitrary waveform generator, which is integrated in the back end. Additionally, to allow the easy control of several units, each back-end unit is controlled/configured over a TCP/IP connection.

The front end comprises an active frequency multiplier, which produces the actual frequency range that probes the plasma, the antenna and signal detection device, which interface the plasma, and either a mixer or a single-end detector. We chose to multiply the back-end frequencies by 6 since this brings us to the 70.8–112.8 GHz frequencies (NATO W bands). The choice for this specific front-end was driven by the legacy of the successful application of this sampling technique in the VUT-1 shock tube [26] for low-ionization shocked flows. Selecting higher frequencies might be more appropriate for typical atmospheric entry conditions (see Figure 10), as the 70.8–112.8 GHz band is likely to be below the cutoff frequency of most shocked flows of interest. On the other hand, front-end developments for higher frequencies incur steeply increasing costs. Moreover, these high-frequency front ends are more difficult to test with steady-state plasma sources since very-high-density plasmas are required. The development of higher-frequency front ends is therefore contingent on the results obtained using the selected frequency band. We note that this equipment could also be considered for probing integrated electronic densities in steady-state plasma wind tunnels. Here, the issue lies in our probing frequencies being significantly above the plasma cutoff frequency, for which the measurements of the phase shift (see Equation (10)) will be less accurate (with phase shifts of a few degrees at most). To offset this issue, a lower-frequency front end working in the 4–18 GHz range (essentially with no frequency multiplication) is under development.

With this architectural choice, a standard compact back end that may connect to different front-end units will enable quick instrument re-configuration to probe different plasma scenarios. Ultimately, several back-end units, along with corresponding front ends, may be run in parallel for probing vaster plasma density regions, simultaneously. This type of modular design shares the hardware components with a reflectometer and can be modified into one if needed.

### 3.5. Proof-of-Concept Application to a Steady-State Plasma Source

**Figure 18:**
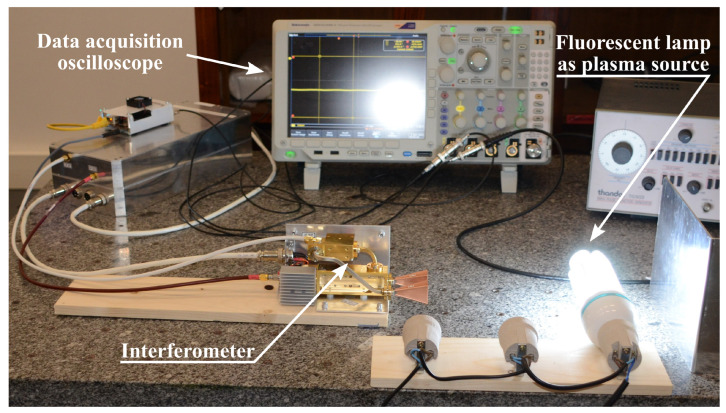
Setup for proof-of-concept experiment.

A fluorescent lamp was used as a plasma source for demonstrating the interferometry concept. The lamp was placed between the emitter/receptor antennas and a metallic plate, where the electromagnetic waves are reflected. With the lamp in the off position, the received signal has a given phase corresponding to the path that the electromagnetic wave travels over the air and inside the lamp, then back inside the lamp and the air upon bouncing on the metallic plate. Once the lamp is turned on, the electromagnetic wave is further delayed, owing to the presence of free electrons inside the lamp, leading to a phase shift in the signal, according to Equation (10). The setup is shown in Figure 18. Data acquisition is performed using a digital oscilloscope.

The signal frequency was set to 70.8 GHz, corresponding to a wavelength of 4.24 mm. The plasma thickness *D* inside the lamp tube was calculated, taking the tube diameter $D_{tube}$ and subtracting its thickness $t_{tube}$. Since the microwave crosses *N* lamp tubes, the result was multiplied by the 2 × *N* to account for the reflection at the metallic wall and round trip as shown in Equation (12):

(12) $$D=2N\left(D_{tube}-2\times t_{tube}\right)=2\times 4\times \left(11.4-2\times 1.4\right)=68.8\;\mathrm{mm}.$$

To convert the voltage signal into an angular phase signal, first the full amplitude (2π rad) of the phase signal needs to be known. This is estimated by performing a frequency sweep on the setup and measuring the full amplitude of the signal, yielding 2π rad≡670 mV.

**Figure 19:**
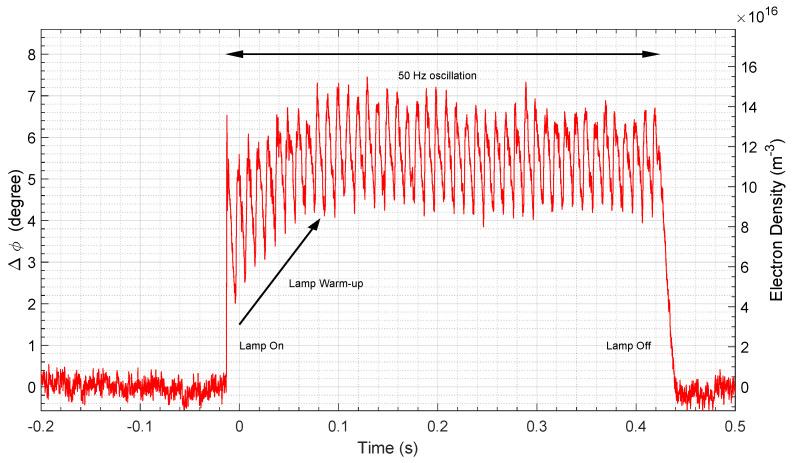
Interferometer phase difference and electron density for the fluorescent lamp plasma source setup.

Figure 19 shows the phase difference in relation to base value and the corresponding average electron density. At t≈−0.02 s, the lamp is turned on, leading to a sharp rise in the voltage signal, and turned off at t≈0.42 s. Once the lamp is turned on, the plasma density starts to increase until a steady state is reached at t≈0.1 s. During lamp operation, an oscillation of the signal is present, which corresponds to the electric grid outlet frequency of 50 Hz.

Computing the phase difference in radians, and then applying Equation (11), since we verify the condition D/λ>3, we obtain an average electron density of $1.2\times 10^{17}$ m${}^{-3}$. The results are on the same order of magnitude as the ones observed by Liu in [101], where the author performed a similar experiment to measure the influence of electric frequency on the electron density of the fluorescent lamps.

This simplified experiment highlights the simplicity and flexibility of this non-intrusive technique for measuring plasma electron densities. The tested configuration with the emitting and receiving antenna placed side by side allow for seamless use of the equipment in any arbitrary plasma source, provided it has two facing windows with the front end placed in front of one and a metallic plate placed in front of the other. Obviously, this requires that a careful alignment is performed to ensure that the electromagnetic waves are properly reflected in the metallic plate and not elsewhere. An alternative would be to decouple the receiving antenna, which would be placed facing the emitting one; however, this requires a bespoke configuration adapted for each plasma source (a specific configuration of this type will be used for measurements in ESTHER). Another limitation is that a plasma source needs to have a size of the same order of magnitude or bigger than the horn antennas to ensure that most of the emitted electromagnetic waves effectively cross the plasma. Lastly, the range of frequencies preferentially needs to be “compatible” with the application as discussed in Section 2.4. The frequency should be higher than the plasma cutoff frequency, as otherwise the wave will not propagate, yet it should not be so high that the phase shift becomes as small as the background noise. With this said, this small experiment shows that reasonable measurements of plasma densities may still be achieved with just a few degrees of phase shift.

Microwave electron density measurements may be cross checked with other techniques, specifically Stark broadening, which is the only other diagnostic which may be straightforwardly applied to shock tubes. To properly assess the applicability range of Stark-broadening techniques, two 1D shock wave simulations were ran, yielding the chemical conditions behind the shock wave for a high-ionization case (Jupiter entry) and a low-ionization case (Mars entry). The broadening of the H-α line was then computed from the post-shock conditions for peak electron density. Table 10 presents the expected broadening of the H-α spectral line caused by different broadening mechanisms. A more detailed description of these is found in [20,39,40]. The total spectral broadening may be modeled as a convolution of the Lorentzian and the Doppler broadening. For the Jupiter entry case, the high entry velocity leads to a post-shock temperature in the vicinity of 42,000 K, and a corresponding electron density of about 1.40 $\times \;10^{21}$ electron/m${}^{3}$. In the case of a Mars entry, the peak electron density is 1.74 $\times \;10^{17}$ electrons/m${}^{3}$ at a temperature of around 3200 K. In the former case, the Lorentz component of the broadening is dominated by the Stark effect, whereas in the latter, the collisional term is dominant. As expected, for lower ionization flows, the Stark line broadening effects are not dominant, meaning interferometry measurements are more advantageous. For highly ionized flows, where the plasma cutoff frequencies require THz-rated diagnostics, Stark-broadening techniques might be more straightforward.

## 4. Conclusions

High-speed events, such as planetary entry shock waves, are very challenging to examine in shock tube facilities, owing to their very short timescales (in the order of the μs), hence mandating the deployment of fast diagnostic techniques. ESTHER is a new state-of-the-art facility designed to reproduce and characterize high-speed entry flows (>10 km/s) by means of spectroscopy and microwave interferometry. The importance of examining the different spectral regions lies in characterizing the physical and chemical processes governing the behavior of the entry plasmas to perfect the numerical models. The instrumentation setups of ESTHER for each of the spectral regions of UV-VUV, visible and NIR/MWIR are discussed. Following a successful application of this concept in the older VUT-1 shock tube, a microwave interferometry setup was developed to enable time-resolved electron-density measurements in ESTHER. An initial proof-of-concept test was conducted, and its results show good agreement with the bibliography review.

As ESTHER is now finally coming to its first operational tests, after a long-winded development cycle of about a decade, it is expected that the array of diagnostics described here will act as an enabler for high-level science and technology obtained in the facility (and other analogues), allowing to shed light on one of the final frontiers of fluid mechanics: aerothermodynamics.

## Figures and Tables

**Table 1:** STAGG parameters for Earth (N${}_{2}$-O${}_{2}$) performance map simulation. Driver input conditions and STAGG running mode (optimization of compression tube pressure or simple computation with fixed conditions).

Mixture Molar Ratios X:H_2_:O_2_	Driver Pressure Filling-Peak (bar)	Driver Post-Combustion Temperature (K)	STAGG Conditions	Shock Wave Velocity (km/s)
N_2_ 10:2:1.4	10–38	1250	Optimized	4.0–5.1
N_2_ 10:2:1.4	30–114	1250	Optimized	4.6–5.5
N_2_ 10:2:1.4	50–190	1250	Optimized	4.8–5.8
He 8:2:1.6	5–30	2650	De-tuned	5.8–10.0
He 8:2:1.6	10–60	2650	De-tuned	6.7–10.8
He 8:2:1.6	5–30	2650	Optimized	7.3–10.2
He 8:2:1.6	10–60	2650	Optimized	8.0–10.9
He 8:2:1.2	10–61.5	2650	Optimized	8.2–11.0
He 8:2:1.2	50–334	2650	Optimized	10.3–13.2
He 8:2:1.4	100–660	2650	Optimized	11.1–13.8

**Table 2:** ESTHER spectroscopy specifications and requirements.

UV-VUV Spectroscopy					
**Equipment**	**Spectral Range**	**Resolution**	**Accuracy**	**Integration Time**	**Signal to Noise Ratio**
Spectrometer + Streak Camera	80–350 nm	0.1 nm @ 110 nm	0.01 nm	≤ 1 μs	>20
**MWIR Spectroscopy**					
**Equipment**	**Spectral Range**	**Resolution**	**Accuracy**	**Integration Time**	**Signal to Noise Ratio**
Spectrometer + iCCD camera	1–6 μm	0.5 nm @ 2.7 μm	0.05 nm	≤ 1 μs	>20

**Table 3:** Comparison between different optical window materials for VUV spectroscopy.

Material	Knoop Hardness	Advantages	Disadvantages
Lithium Fluoride	100 Kg/mm^2^	Large VUV-IR transmittance	Very brittle, expensive
Magnesium Fluoride	415 Kg/mm^2^	Large VUV-IR transmittance	Brittle, expensive
VUV graded Sapphire	1370 Kg/mm^2^	Cheap, resistant, some VUV capabilities	Limited transmittance window
Fused Quartz	741 Kg/mm^2^	Cheap, resistant	Limited transmittance window

**Table 4:** CFD simulation conditions and parameters. All simulations 1D post-shock relaxation with initial gas temperature of 300 K.

Planetary Object	Chemical Mixture	Pressure (Pa)	Velocity $v_{\infty }$ (km/s)	Chemical Model	Reference
Earth	N${}_{2}$-O${}_{2}$ (79-21%)	26.66	10.29	[91]	[45,92]
Mars	CO${}_{2}$-N${}_{2}$ (95-5%)	57	2.6	[91]	[93]
Venus	CO${}_{2}$-N${}_{2}$ (96.5-3.5%)	447	8.9	[91]	[94]
Venus	CO${}_{2}$-N${}_{2}$ (96.5-3.5%)	37	10.6	[91]	[94]
Titan	N${}_{2}$-CH${}_{4}$ (95-5%)	13.3	5.15	[95]	[94]
Neptune	H${}_{2}$-He-CH${}_{4}$ (79.75-18.7-1.54%)	892	18.3	[36]	[36]
Jupiter	H${}_{2}$-He (89-11%)	27.5	46.7	[96]	[36]

**Table 5:** Calibration results for the 1200 g/mm and 600 g/mm gratings, Hg 253.65 nm line, with FWHM in pixels.

1200 g/mm			600 g/mm		
Position	Resolution (Pixel)	Resolution (nm)	Position	Resolution (Pixel)	Resolution (nm)
Left	28	0.36	Left	18.9	0.47
Center	17.8	0.23	Center	24.6	0.61
Right	25.1	0.32	Right	46.8	1.17

**Table 6:** ESTHER spectroscopy acceptance campaign results.

–	Resolution 1200 g/mm	Resolution 600 g/mm	Accuracy	Signal-to-Noise Ratio
Specifications and Requirements	0.25 nm	0.50 nm	5 pixels	>100
Test	0.23 nm @ 253.65 nm	0.47 nm @ 253.65 nm	4.5 pixels	3425

**Table 7:** Infrared spectroscopy optical fiber pre-selection equipment.

Optical Fiber	Material	Spectral Range (μm)	Core Diam. (μm)	Attenuation dB/m	Transmissivity
Thorlabs MF22L2	InF${}_{3}$	0.310–5.5	200	<0.25 [2–4 μm]	88%
Thorlabs MZ22L2	ZrF${}_{4}$	0.285–5.5	200	<0.2 [2–3.5 μm]	91%
Le Verre Fluoré IFG MM 200/260	InF${}_{3}$	0.310–5.5	200	<0.01 @ 3.5 μm	>81%
Guiding Photonics Mid-IR	Glass	2–16	200	4	45%
Art Photonics CIR250/300	Chalcogenide	1–5.5	250	0.3 [1–4 μm]	42%

**Table 8:** Infrared spectroscopy spectrograph pre-selection equipment.

Spectrograph	Focal Length	Aperture Number	Grating [Blazing]	Resolution (nm)
McPherson 2035	350 mm	*f*/4.8	300 g/mm [2 μm]	0.2 @ 312.6 nm
			20 g/mm [3.7 μm]	3 @ 312.6 nm
McPherson 207	670 mm	*f*/4.7	300 g/mm [2 μm]	0.16 @ 312.6 nm
			20 g/mm [3.7 μm]	2.04 @ 312.6 nm
McPherson 2061	1000 mm	*f*/7	300 g/mm [2 μm]	0.07 @ 312.6 nm
			20 g/mm [3.7 μm]	1.05 @ 312.6 nm
McPherson 209	1330 mm	*f*/4.7	300 g/mm [2 μm]	0.04 @ 312.6 nm
			20 g/mm [3.7 μm]	0.6 @ 312.6 nm
Princeton HRS-300	300 mm	*f*/3.9	300 g/mm [2 μm]	0.4 @ 500 nm
			50 g/mm [0.6 μm]	2.4 @ 500 nm
Princeton HRS-750	750 mm	*f*/9.7	300 g/mm [2 μm]	0.16 @ 500 nm
			50 g/mm [0.6 μm]	0.96 @ 500 nm

**Table 9:** Infrared spectroscopy fast iCCD cameras characteristics.

Fast Infrared Camera	Aperture Number	Resolution	Min. Int. Time (μs)	NETD (mK) *
Flir X8580	*f*/2.5	1280 × 1024	0.27	⩽30
Flir X6980	*f*/2.5	640 × 512	0.27	⩽30
Infratec Image IR 9400	*f*/2.2	640 × 512	0.10	<30 @ 30 °C
Telops FAST M1K	*f*/2.5	640 × 512	0.27	⩽25
Telops FAST M200hd	*f*/3	1280 × 1024	0.50	⩽20
Telops FAST M100hd	*f*/3	1280 × 1024	0.50	⩽20
Tigris 640 InSb BB	*f*/3	640 × 512	NA	⩽25

* The noise equivalent temperature different (NETD) is the parameter that regulates the signal photon resolution.

**Table 10:** Discrimination of the different spectral broadening effects for shock tube experiments on the H−α line (656.46 nm). Data were computed from the simulations in Table and using SPARK-LbL code [39].

Case	FWHM Broadening (cm^−1^)					
	Collisional	van der Waals	Resonance	Stark	Lorentz (All)	Doppler
Jupiter	0.056	0.011	0.173	0.522	0.762	2.228
Mars *	0.551	0.068	0.006	0.004	0.630	0.713

* Mars conditions adapted from VUT-1 shock tube test case [64]: pure CO${}_{2}$, $v_{\infty }=3.4$ km/s, $p_{\infty }=826.5$ Pa.

## Data Availability

Not applicable.

## Abbreviations

The following abbreviations are used in this manuscript:

CFD	Computational Fluid Dynamics
EAST	Electric Arc Shock Tube
ESA	European Space Agency
ESTHER	European Shock Tube for High Enthalpy Research
FWHM	Full Width at Half Maximum
HVST	Hyper Velocity Shock Tube
iCCD	Intensified Charge Coupled Device
IPFN	Instituto de Plasmas e Fusão Nuclear
IST	Instituto Superior Técnico
MWIR	Mid-Wavelength Infrared
NETD	Noise Equivalent Temperature Different
NIR	Near Infrared
SPARK	Software Package for Aerothermodynamics Radiation and Kinetics
STAGG	Shock Tube And Gas Gun
UV	Ultraviolet
VCO	Voltage Controlled Oscillator
VUV	Vacuum Ultraviolet
